# Plasma Levels of α-Synuclein, Aβ-40 and T-tau as Biomarkers to Predict Cognitive Impairment in Parkinson’s Disease

**DOI:** 10.3389/fnagi.2020.00112

**Published:** 2020-04-28

**Authors:** Nai-Ching Chen, Hsiu-Ling Chen, Shau-Hsuan Li, Yen-Hsiang Chang, Meng-Hsiang Chen, Nai-Wen Tsai, Chiun-Chieh Yu, Shieh-Yueh Yang, Cheng-Hsien Lu, Wei-Che Lin

**Affiliations:** ^1^Department of Neurology, Kaohsiung Chang Gung Memorial Hospital, Chang Gung University College of Medicine, Kaohsiung, Taiwan; ^2^Department of Diagnostic Radiology, Kaohsiung Chang Gung Memorial Hospital, Chang Gung University College of Medicine, Kaohsiung, Taiwan; ^3^Department of Oncology and Hematology, Kaohsiung Chang Gung Memorial Hospital, Chang Gung University College of Medicine, Kaohsiung, Taiwan; ^4^Department of Nuclear Medicine, Chang Gung University College of Medicine, Kaohsiung Chang Gung Memorial Hospital, Kaohsiung, Taiwan; ^5^MagQu Co., Ltd., New Taipei City, Taiwan

**Keywords:** Parkinson’s disease, immunomagnetic reduction, plasma biomarker, plasma Aβ-40, Aβ-42, T-tau, α-synuclein

## Abstract

**Objective:**

In this study, we assessed plasma biomarkers to identify cognitive impairment in Parkinson’s disease (PD) patients by applying ultra-sensitive immunomagnetic reduction-based immunoassay (IMR).

**Methods:**

The study enrolled 60 PD patients and 28 age- and sex-matched normal controls. Complete cognitive function assessments were performed on participants using the Mini-Mental State Examination (MMSE) and Clinical Dementia Rating. PD patients with an MMSE score of ≦26 were defined as having cognitive impairment. Meanwhile, a 99mTc-TRODAT-1 scan was performed and plasma levels of Aβ-40, Aβ-42, T-tau, and α-synuclein were evaluated using IMR, subsequent correlation analyses were then performed.

**Results:**

Compared with normal adults, PD patients have higher plasma levels of α-synuclein and T-tau, and a lower level of Aβ-40 (*p* < 0.05). Plasma levels of α-synuclein (*r* = −0.323, *p* = 0.002), Aβ-40 (*r* = 0.276, *p* = 0.01), and T-tau (*r* = −0.322, *p* = 0.002) are significantly correlated with MMSE scores. The TRODAT scan results, including visual inspection and quantification, revealed significant correlations between Aβ-40 and PD. Multiple regression analysis showed that the plasma levels of Aβ-40 (OR = 0.921, 95% CI = 0.879–0.962), α-synuclein (OR = 3.016, 95% CI = 1.703–5.339), and T-tau (OR = 1.069, 95% CI = 1.026–1.115) were independently associated with PD patients with cognitive impairment. The cutoff values for predicting cognitive deficits in PD patients were 45.101 pg/ml of Aβ-40, (Area under curve (AUC) = 0.791), 0.389 pg/ml of α-synuclein, (AUC = 0.790), and 30.555 pg/ml of T-tau (AUC = 0.726).

**Conclusion:**

Plasma levels of α-synuclein, Aβ-40, and T-tau are potential biomarkers to detect cognitive impairment in PD patients.

## Introduction

Parkinson’s disease (PD) is a serious neurodegenerative disorder commonly regarded as influencing patients’ motor system. However, further increasing the disease burden and decreasing quality of life are multiple non-motor symptoms affecting almost all PD patients, which include cognitive impairment, psychiatric and sleep disorders, autonomic dysfunction, and sensory abnormalities (Simuni and Sethi, 2008). In aging societies worldwide, significant economic burdens related to healthcare and non-healthcare costs will increasingly be incurred for the management of patients with PD and PD patients with dementia (PDD). Therefore, the Movement Disorders Society (MDS) has conducted several studies to develop a screening checklist to facilitate early detection of PDD (Barton et al., 2012; Serrano-Duenas et al., 2017; Skorvanek et al., 2018).

There is substantial evidence indicating that the conversion of α-synuclein from soluble monomers to aggregated, insoluble forms in the brain is a pathognomonic finding in PD patients. This mechanism may also be found in human bodily fluids, such as cerebrospinal fluid (CSF) and blood plasma (El-Agnaf et al., 2003; Martin et al., 2004). As no single biomarker is known which can detect and correlate with motor dysfunction severity or cognitive impairment in PD patients, the diagnosis of PD currently relies heavily upon clinical criteria (Gelb et al., 1999). Furthermore, the effectiveness of laboratory testing in the initial diagnosis and subsequent evaluation of disease progression remains limited. In addition to α-synuclein, levels of CSF Aβ-38, Aβ-40, and Aβ-42 are altered in early stage PD patients, and are associated with memory impairment (Alves et al., 2010). A meta-analysis report reviewing 16 studies of CSF biomarkers and cognitive impairment in PD patients concluded that both amyloid pathology and tauopathy could be involved in the development of dementia in PD patients, similar to Alzheimer’s disease (AD) (Hu et al., 2017). However, studies investigating plasma biomarkers and correlations to cognitive decline in PD patients remain lacking.

At present, CSF, serum, or plasma biomarkers are primarily analyzed with immunoassay techniques such as ELISA; however, these assays are often performed manually which makes them challenging to standardize, and often results in substantial measurement variabilities. Previous studies have reported on the application of a sensitive immunomagnetic reduction-based immunoassay (IMR), which can quantitatively detect plasma biomolecules of Aβ-40, Aβ-42, and T-tau at ultra-low concentrations for the early detection of cognitive impairment (Yang et al., 2011; Chiu et al., 2014). Meanwhile, plasma α-synuclein analysis performed with IMR has been shown sensitive enough to effectively discriminate patients with PD or PDD (Yang et al., 2016; Lin et al., 2017). With proven IMR sensitivity, and the availability of blood plasma samples, it is reasonable to suggest these may facilitate measurement of plasma α-synuclein, Aβ-40, Aβ-42, and T-tau from both normal control subjects and PD patients.

As the cognitive continuum applied in the evaluation of PD patients, ranging from normal cognition to cognitive impairment, lacks clearly defined stages, identification of potential biomarkers to evaluate degrees of PD cognitive impairment is critical. For this study, we employed an IMR-based immunoassay to systematically investigate the complex associations between multiple proteinopathies, specifically plasma α-synuclein, Aβ-40, Aβ-42, and T-tau, as well as the cognitive and motor function status of PD patients and controls.

## Materials and Methods

### Subjects

For this study, 60 PD patients were prospectively enrolled at the Neurology Department of Chang Gung Memorial Hospital. A qualified neurologist made a definitive clinical diagnosis of each patient. Only those patients with a definitive diagnosis of idiopathic PD according to the criteria of bradykinesia, meaning slowness of initiation of voluntary movement with progressive reduction in speed and amplitude of repetitive actions, and one of following presentations: (1) muscular rigidity, (2) 4-6 Hz rest tremor, (3) posture instability not caused by primary visual, vestibular, cerebellar, or proprioceptive dysfunction, were included in the study (Hughes et al., 1992). Disease severity and functional status of patients were assessed by the modified Hoehn and Yahr staging scale (HY-stage) (Goetz et al., 2004), and Unified Parkinson’s Disease Rating Scale (UPDRS) (Gasser et al., 2003). Meanwhile, 28 sex- and age-matched healthy subjects without medical history of neurological disease, alcohol or substance abuse, psychiatric illness, or head injury, and with similar education levels were enrolled in the study for controls. To investigate the effects of disease stage on the change in plasma biomarkers, we further divided the PD patients into two subgroups: PD patients without cognitive impairment and PD patients with cognitive impairment. The study protocol was approved by the ethics committee of Chang Gung Memorial Hospital. Written informed consent was provided by all study participants or their guardians prior to enrollment in the study (CGMH-IRB No. 201802352A3B0).

### Neurobehavioral Evaluation and Cognitive Severity Definition

The Mini-Mental State Examination (MMSE) was used to evaluate general intellectual functions (Folstein et al., 1975). Since it scores functional capacity of the participant independent of physical disability, cognitive severity was defined using the Clinical Dementia Rating (CDR) (Schafer et al., 2004). An experienced neuropsychiatrist determined the CDR after a face-to-face, semi-structured interview with the patient and a dependable caregiver. In recognition of the importance of cognitive impairment in PD, the Movement Disorder Society published recommendations for the diagnosis of dementia in PD patients in 2007 (Dubois et al., 2007). The MMSE was recommended as a primary clinical tool for PD dementia. Therefore, in the present study we defined patients with MMSE scores of ≦26 as PD-with cognitive impairment (Burdick et al., 2014).

### Blood Sampling and Assaying of Plasma Biomarkers: Plasma Aβ-40, Aβ-42, T-tau, and α-Synuclein

To avoid the sleep-wake cycles in plasma biomarkers, we collected blood for all participants at a fixed time interval between 10:00 AM-11:30AM. Blood was not collected within 2 h post exercise. The blood draw was performed using a 10-ml K3-EDTA tube (Greiner Bio-One 455036), followed by gently inverting the tube 10 times immediately after blood draw. The centrifugation was conducted at 15–25°C and 1500–2500 *g* for 15 min by using a swing-out (bracket) rotor. Subsequently, 0.5 ml of plasma (supernatant) was removed from the blood tube and transferred into a fresh 1.5 ml Eppendorf tube. The aliquoted plasma samples were frozen at −80°C within 3 h after blood draw before performing assays.

The methodology is detailed in our previous studies (Yang et al., 2016; Lin et al., 2017; Yang et al., 2017; Lin et al., 2018; Yang et al., 2018). Various IMR kits were used to separately assay concentrations of Aβ-40 (MF-AB0-0060, MagQu), Aβ-42 (MF-AB2-0060, MagQu), T-tau (MF-TAU-0060, MagQu), and α-synuclein (MF-ASC-0060, MagQu) in human plasma. For assaying Aβ-40, α-synuclein, and T-tau, 80-μl reagent was mixed with 40-μl plasma. For assaying Aβ-42, 60-μl reagent was mixed with 60-μl plasma. The IMR analyzer (XacPro-S) was then utilized to identify IMR signals, which were transformed to biomarker concentrations via the concentration-dependent IMR signal (Yang et al., 2016; Lin et al., 2017; Yang et al., 2017; Lin et al., 2018; Yang et al., 2018). For each biomarker assay, measurements were performed in duplicate. The averaged value of the duplicated measurements was used to show the detected concentration of a biomarker. The ratio, referred to as CV%, of standard deviation to averaged value of the duplicated measurements was then calculated. The accepted CV% was below 20% for Aβ-40, Aβ-42, and T-tau, while accepted CV% was below 25% for α-synuclein. Once CV% was higher than 20% or 25%, one more measurement was done. Two of the triple measurements showing CV% below 20% or 25% were then selected as duplicated measured concentrations. The averaged value of the duplicated measurements was used to show the detected concentration of a biomarker.

### TRODAT-1 Acquisition and Quantitative Analysis

All patients were injected intravenously with a single bolus dose of 925 MBq (25 mCi) 99mTc-TRODAT-1. Brain images were subsequently obtained after 4 h using a hybrid SPECT/CT system (Symbia T; Siemens Medical Solutions, Hoffman Estate, IL, United States). The SPECT/CT scanner was equipped with low-energy, higher solution collimators, and a dual-slice spiral CT. The SPECT acquisition parameters were a 128 × 128 matrix with 60 frames (40 s/frame); while scan parameters for the CT were 130 kV, 17 mA, 5-mm slices, and an image reconstruction with a medium-smooth kernel. SPECT images were attenuation-corrected based on CT images, and scatter-corrected with Flash 3-dimensional (3D) algorithm (ordered subsets expectation and 3D maximization with resolution correction) with 8 subsets and 8 iterations.

For quantification of 99mTc-TRODAT-1 binding in striatum, the specific-to-non-specific binding ratio was calculated using the summation of 3 adjacent transversal slices representing the highest intensity striatal DAT binding. We applied the low DAT concentration area of the occipital cortex as a background ROI. The principal investigator determined the ROI delineation according to the CT portion of SPECT/CT images (Hsu et al., 2014). The left and right striatal uptake ratio (SUR) was calculated using the following formula:

SUR=[(striatalcounts)/(striatalROI)/(occipitalcortexcounts)/(occipitalcortexROI)]

To classify the 99mTc-TRODAT-1 uptake of striatum by visual inspection, a 5-point scoring system was used and defined as follows: 0 = normal striatal uptake; 1 = normal caudate but decreased putamen uptake at its tail portion; 2 = normal caudate but no putamen uptake; 3 = decreased caudate uptake with no putamen uptake; 4 = total loss or near total loss of striatal uptake. Scores of left and right striatum were analyzed separately. All the 99mTc-TRODAT-1 SPCET/CT scans were interpreted using the consensus of two experienced nuclear medicine attending physicians with no knowledge of the data from the clinical studies.

### Statistics

All values are expressed as mean ± standard deviation or median (interquartile range). Chi-square test was used for categorical variable comparison. The normality of distribution was tested by Kolmogorov–Smirnov test. Non-parametric methods were used when continuous variables were non-normal distribution or for small sample size. Mann–Whitney U tests were applied to compare the difference between the two groups. The Jonckheere-Terpstra test was used to access trends in the 4 plasma biomarkers (α-synuclein, T-tau, Aβ-40, and Aβ-42 levels) with increasing variables (CDR, CDR sum of box, Modified Hoehn and Yahr scale, TRODAT visual scale included right and left). Spearman rank correlation was used to determine relationships between plasma biomarkers and clinical severity, including cognitive severity and PD. Simple and multiple binary logistic regression were used to determine the factors associated with cognitive impairment in PD. The area under the curve (AUC) of the receiver-operator curves (ROC) for three plasma biomarkers was calculated to measure the predictive value of cognitive impairment in PD patients. The best cut-off points were found according to maximum Youden’s index (sensitivity + specificity-1). Statistical analysis was performed using the SPSS Version 22.0 software package. A *p*-value < 0.05 was considered statistically significant.

## Results

### Clinical Characteristics

The characteristics and specific demographic data of the 60 patients with PD and 28 controls are listed in Table 1. The mean age of the PD group had no significant difference from the controls (62.8 ± 9.1 vs. 61.1 ± 4.9, *p* = 0.253). The PD group (21 men, 39 women) and the control group (8 men, 20 women) also had no significant difference in gender. Meanwhile, PD patients had significantly lower MMSE score and higher CDR, compared with controls.

**TABLE 1:** Clinical characteristics in Parkinson’s disease patients.

	Normal control (*n* = 28)	PD (*n* = 60)	
Gender (male/female)	8/20	21/39	0.619
Age (years)	61.1 ± 4.9	62.8 ± 9.1	0.174
Cognitive impairment (%)*	0	78%	<0.001
Disease duration (years)	N.A.	1 (0.0, 3.0)	
MMSE*	29.0 (28.0, 29.0)	26 (22.0, 27.0)	<0.001
CDR*	0	0.50 (0.00, 0.50)	<0.001
CDR sum of box*	0	0.50 (0.00, 2.00)	<0.001
Modified Hoehn and Yahr scale, mean	N.A.	2.00 (0.00, 2.50)	
UPDRS I, mean	N.A.	3.00 (2.00, 5.00)	
UPDRS II, mean	N.A.	9.00 (6.00, 12.00)	
UPDRS III, mean	N.A.	20.00 (16.00, 34.00)	
UPDRS total, mean	N.A.	32.00 (23.00, 48.00)	
α-synuclein (pg/ml)*	0.06 (0.02, 0.08)	1.35 (0.88, 1.98)	<0.001
Aβ1-40 (pg/ml)*	56.45 (48.78, 65.05)	35.73 (31.71, 41.83)	0.018
Aβ1-42 (pg/ml)	14.37 (13.42, 17.91)	18.05 (16.00, 21.60)	0.539
T-Tau (pg/ml)*	14.67 (11.02, 22.48)	31.87 (26.06, 3733)	<0.001

Mean ± standard deviation or median (interquartile range). In clinical characteristics, gender was analyzed by using chi-square. Age, MMSE, CDR and CDR sum of box were analyzed by using the Mann–Whitney U test. PD = Parkinson’s disease; UPDRS = The Unified Parkinson’s Disease Rating Scale; MMSE = Mini-Mental State Examination; CDR = Clinical Dementia Rating; * = *P* < 0.05 vs. normal control.

According to a previous report, the sensitivity of the MMSE≦26 to detect dementia was 45% in a subset of participants who underwent clinical diagnostic procedures (Burdick et al., 2014).

In terms of the cognitive function evaluation, the PD patient group with normal cognitive function was defined as MMSE > 26 (*n* = 20), and PD-with cognitive impairment was defined as MMSE ≦26 (*n* = 40). The mean UPDRS part I, II, III scores, and Modified Hoehn and Yahr scale are detailed in Table 1. The study revealed plasma levels of α-synuclein to be significantly higher in PD patients (median = 1.35) compared with controls (median = 0.06). To address the possibly confounding influence of age on α-synuclein plasma concentrations, we analyzed the plasma α-synuclein levels in control subjects of various ages, revealing no significant correlation between plasma α-synuclein levels and age in control subjects (correlation coefficient *r* = −0.098, *p* = 0.620). In addition, the study found plasma levels of Aβ-40 to be significantly lower in patients with PD (median = 35.73) compared with controls (median = 56.45). Plasma levels of Aβ-42 exhibited no significant difference in patients with PD compared with normal controls; however, plasma levels of T-tau were noted to be significantly higher in PD patients (median = 31.87) compared with controls (median = 14.67).

### Correlations Between Plasma Biomarkers, Cognitive Function, and PD Severity

The study demonstrated that plasma levels of α-synuclein, T-tau, Aβ-40, and Aβ-42 were significantly correlated with scores of MMSE, CDR, CDR sum of box, UPDRS parts I, II, III, and total scores, and Modified Hoehn and Yahr scale. The correlation analysis was performed between 99mTc-TRODAT-1 and the plasma levels of Aβ-40, T-tau, and α-synuclein, revealing that only Aβ-40 plasma level had correlations with the right visual inspection scale (*r* = −0.315, *p* = 0.048), and right SUR (*r* = 0.321, *p* = 0.044) (Table 2).

**TABLE 2:** Correlation between cognitive function, Parkinson’s disease severity and plasma biomarkers.

	α-synuclein	Aβ1-40	Aβ1-42	T-Tau
**Cognitive test**				
Mini-Mental State Examination	−0.283**	0.263*	–0.159	−0.213*
Clinical Dementia Rating	0.411**	−0.355**	0.337**	0.298**
Clinical Dementia Rating sum of box	0.384**	−0.345**	0.250*	0.325**
**Parkinson’s disease severity**				
Modified Hoehn and Yahr scale, mean (S.D.)	0.620**	−0.477**	0.331*	0.545**
UPDRS I, mean (S.D.)	0.274*	−0.360**	0.150	0.228*
UPDRS II, mean (S.D.)	0.417**	−0.411**	0.213*	0.281**
UPDRS III, mean (S.D.)	0.390**	−0.419**	0.217*	0.289**
UPDRS total, mean (S.D.)	0.399**	−0.427**	0.217*	0.292**
**TRODAT**				
Right visual grading	0.003	−0.315*	0.134	–0.170
Left visual grading	0.012	–0.295	0.049	–0.088
Right SUR ratio	–0.110	−0.321*	0.192	0.223
Left SUR ratio	–0.140	–0.273	0.140	0.127
Mean ratio	–0.130	–0.308	0.173	0.182

UPDRS = The Unified Parkinson’s Disease Rating Scale, SUR = striatal uptake ratio, CDR = Clinical Dementia Rating, MMSE = Mini-Mental State Examination, Spearman rank correlation analysis to determine relationship between plasma biomarkers and cognitive test, Parkinson’s disease severity and TRODAT. ** = *p* < 0.01; * = *p* < 0.05.

### Binary Logistic Regression for Biomarkers Related to Cognitive Impairment in PD Patients (Table 3)

Variables including age, plasma levels of Aβ-40, Aβ-42, T-tau, and α-synuclein were applied for binary regression analysis to identify significant predictors associated with cognitive impairment (those with MMSE scores ≦26) in PD patients. Multiple binary logistic regression with stepwise selection was performed, from which plasma levels of α-synuclein and Aβ-40 were revealed to be independently associated with cognitive impairment in patients with PD. We further performed a correlation analysis between the four biomarkers, revealing moderate to high correlations between the four biomarkers (Supplementary Table 1). After adjustments for age, plasma levels of Aβ-40, T-tau, and α-synuclein were revealed to be independently associated with cognitive impairment in patients with PD.

**TABLE 3:** Binary logistic regression models for cognitive impairment in Parkinson’s disease patients.

	Simple			Multiple		
Variables	OR	95% C.I.	*P*-value	OR	95% C.I.	*P*-value
α-synuclein (pg/ml)	3.02	1.70–5.34	<0.001	2.41	1.36–4.26	0.003
Aβ1-40 (pg/ml)	0.92	0.88–0.96	<0.001	0.94	1.36–4.26	0.019
Aβ1-42 (pg/ml)	1.05	0.97–1.13	0.244			
T-Tau (pg/ml)	1.07	1.03–1.12	0.002			

*OR = Odds ratio, C.I. = confidence interval. Multiple binary logistic regression with stepwise selection was performed after adjusting by age.*

### Discrimination of Normal Cognitive Subjects and PD Patients With Cognitive Impairment (Figure 1)

ROC analysis was used to evaluate the plasma levels of Aβ-40 and α-synuclein to discriminate/distinguish between normal cognitive subjects and PD patients with cognitive impairment. The cutoff value between normal cognitive subjects and patients with cognitive impairment was 0.5221 for α-synuclein (sensitivity = 95.0%, specificity = 64.6%, AUC = 0.799, *P* < 0.001); and 30.555 for T-tau (sensitivity = 67.5%, specificity = 77.1%, AUC = 0.726, *P* < 0.001). Due to Aβ-40 levels being higher in normal cognitive subjects, we inverted the number; subsequently, the inverted cutoff value for Aβ-40 between normal cognitive subjects and patients with cognitive impairment was 0.0199 (original level = 50.251, sensitivity = 95.0%, specificity = 47.9%, AUC = 0.697, *P* = 0.002).

**FIGURE 1:**
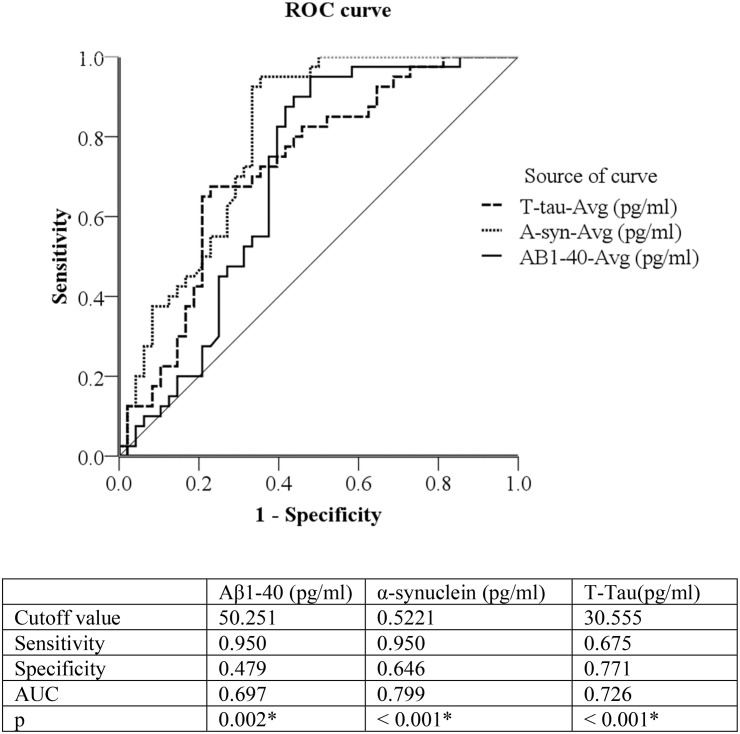
Discrimination of Normal Cognitive function versus PD patients with cognitive impairment by 3 plasma biomarkers: plasma Aβ-40, α-synuclein, and T-Tau (pg/ml). Due to Aβ1-40 being higher in normal controls, we used 1/Aβ1-40 to draw the ROC curve. Aβ1-40 (pg/ml) cutoff value = 50.251, inverted Aβ1-40 (pg/ml) = 0.0199 ROC curve = Receiver operating characteristic curve, PD = Parkinson disease PD patients with cognitive impairment = Mini-Mental State Examination ≦26.

## Discussion

Although CSF biomarkers have demonstrated potential effectiveness for diagnosis of PD with dementia, CSF is acquired through lumbar puncture, which is a relatively invasive procedure compared with obtaining other body fluids, such as blood. This is the first study using plasma biomarkers (α-synuclein, T-tau, Aβ-40, and Aβ-42 levels) with an ultra-sensitive IMR method to determine cognitive impairment in PD patients. We found that three of the four plasma levels differed between PD patients and the control group; specifically, plasma levels of α-synuclein and T-tau were significantly higher, while Aβ-40 was significant lower in PD patients. Furthermore, the four plasma biomarkers exhibited correlations with cognitive evaluations (MMSE and CDR scores) and severity of PD (UPDRS). Finally, multiple regression analysis determined that plasma levels of α-synuclein, Aβ-40 and T-tau are appropriate surrogate biomarkers for identifying cognitive impairment in PD.

The aggregation of α-synuclein with Lewy body formations in the brain as a pathognomonic mechanism in PD patients has led to the belief that this protein plays a central role in a subgroup of neurodegenerative diseases known as synucleinopathies. As it may also be found in other body fluids, such as CSF and blood plasma (El-Agnaf et al., 2003), α-synuclein levels have previously been assessed as biomarkers of PD; nonetheless, an investigation of plasma and saliva α-synuclein levels failed to distinguish PD from normal controls (Goldman et al., 2018). The present study, however, demonstrates that PD patients indeed had a higher plasma α-synuclein level, further correlated with motor severity scores of patients with PD, in line with a previously referenced meta-analysis (Bougea et al., 2019). Furthermore, the plasma α-synuclein level also correlated with cognitive performance, a finding similar to those of previous studies (Lin et al., 2017).

### Roles of Aβ40 and Aβ-42

It has been reported that α-synuclein can interfere with monomeric Aβ-40, with the primary site of interference possibly between amino acid residue positions 12–33 (Chan et al., 2016). The pathophysiology of PD is multifactorial, although misfolded α-synuclein aggregation is considered to be a critical mechanism. Meanwhile, Amyloid-β (Aβ) and tau deposition are also considered comorbid associations, with Aβ deposition in particular being reported to have associations with cognitive decline in PD patients (Jellinger et al., 2002). Furthermore, studies have demonstrated that CSF Aβ is associated with postural instability, gait difficulty, and the newly proposed cholinergic subtype of PD, indicating it is a potential risk factor for cognitive decline in PD patients (Alves et al., 2013; Zuo et al., 2017). This study demonstrates that Aβ-40 have significant positive correlation and Aβ-42 reveal significant negative correlations with cognitive evaluation scores (MMSE and CDR), and severity of PD (UPDRS). One manuscript report similar finding in same IMR Aβ-40 and Aβ-42 as plasma biomarker in to discriminate normal control from patients with Alzheimer dementia. Plasma Aβ-40 was lowest in patient with Alzheimer dementia but Aβ-42 was highest in patient with Alzheimer dementia (Fan et al., 2018). In terms of pathogenesis, Aβs (Aβ1–40 and Aβ1–42) are constantly secreted by many types of cell and are normally found in CSF. Recently, studies have shown that monomeric Aβ1–40 has neuroprotective effects against metal-induced oxidative damage, and Aβ1–42-induced neuronal death (Zou et al., 2002; Zou et al., 2003). For the PD patients in this study, the protective Aβ–40 decreased and neurotoxic Aβ–42 increased. In addition, plasma Aβ-40 was found to be correlated with decreased 99mTc-TRODAT-1 values in patients with PD, reflecting (dopamine) neuron loss, the primary pathologic hallmark of PD (Zhang et al., 2018). This is a complicated process, possibly initiated years or even decades before the clinical onset of the disease, suggesting that detecting soluble misfolded oligomeric forms of abnormal proteins in CSF or blood may provide an effective strategy to identify individuals at risk of developing PD, and to subsequently monitor disease progression in PD patients.

### Role of Tau Proteins

Pathologies and dementias of the nervous system, including AD and PD (Nishimura et al., 2004), are associated with defective tau proteins, no longer capable of properly stabilizing microtubules. Levels of tau neurofibrillary tangles (NFTs), a pathologic hallmark of AD, have an inverse correlation with cognitive performance in patients with PDD (Jellinger et al., 2002; Jellinger, 2007; Jellinger and Attems, 2008). In initial studies, PD was not considered to be a conventional tauopathy. Soluble, unfolded tau, after phosphorylation or mutation, becomes insoluble and misfolded, leading to conformational changes in microtubules and NFTs (Jellinger et al., 2002). Previous imaging studies have demonstrated similar abnormal tau binding topography in patients with dementia with Lewy bodies, both in PD (Gomperts et al., 2016), and AD (Mattsson et al., 2017). In the present study, correlations between high plasma T-tau and cognitive evaluation scores (MMSE and CDR) and severity of PD (UPDRS), further support the role of tau in neuron degeneration. Tau-containing NFTs may act synergistically with α-synuclein pathology to worsen the prognosis both in AD and PD patients.

### Plasma Biomarkers and 99mTc-TRODAT-1

Development of 99mTc-labeled receptor-specific imaging agents to study the central nervous system offer promise for the evaluation of brain function, both in the normal and disease state (Marshall et al., 2006). Clinicopathological correlation studies have suggested that dementia correlates highly with Lewy bodies in certain limbic and cortical areas in PD patients (Huang et al., 2015; Koshimori et al., 2015). Furthermore, TRODAT-1 has identified a correlation between clinical data and abnormalities of the nigrostriatal pathway in PD (Gupta et al., 2019; Patel et al., 2019). Accumulation of protein aggregates is the primary mechanism resulting in cellular dysfunction in neurodegenerative disorders such as AD and PD, which present with similar progressive patterns of neuronal death, nervous system deterioration, and cognitive impairment. The visual and quantification scale 99mTc-TRODAT-1 imaging results herein revealed significant correlations with Aβ-40 in patients with PD, suggesting that Aβ-40 level could be an effective biomarker to detect and monitor the development and progress of PD.

Studies in APP transgenic mice for AD pathogenesis (Manso et al., 2016) indicate critical balances between Aβ deposited in the brain, soluble Aβ in CSF, and Aβ in plasma. More specifically, Tg2576 APP KN670-1ML mice demonstrated age-related Aβ depositions in the brain are associated with reductions of CSF and plasma Aβ levels, presenting difficulties in exploration and learning. In the present study, we found that plasma Aβ-40 is significantly correlated with 99mTc-TRODAT-1 and clinical stages of cognitive impairment in PD patients. Furthermore, after adjustment for age and sex, multiple regression models for cognitive impairment in PD patients revealed Aβ-40 (OR = 0.921, 95%CI = 0.879–0.964), T-tau (OR = 3.016, 95%CI = 1.703–5.339), and α-synuclein (OR = 1.069, 95%CI = 1.026–1.115) to be independently associated with cognitive impairment in patients with PD. The cutoff value for predicting cognitive impairment in PD patients were 50.251 pg/ml of Aβ-40, (AUC = 0.697), 30.555 pg/ml of T-tau, (AUC = 0.726) and 0.522 pg/ml of α-synuclein, (AUC = 0.799). Therefore, after multiple regression analyses, plasma α-synuclein and Aβ-40 demonstrate promise as appropriate surrogate biomarkers for determination of cognitive impairment in PD.

One significant novelty of this study lies in the fact that it is the first to employ an IMR-based method to validate biomarker status. We investigated four plasma biomarkers α-synuclein, T-tau, Aβ-40, and Aβ-42 to distinguish PD patients with cognitive impairment; moreover, clinical evaluations were performed using MMSE, CDR, UPDRS, and 99mTc-TRODAT-1. However, some limitations exist. First, we assessed cognitive functions of study participants using MMSE and CDR, which are relatively simple measurements of cognitive function; whereas, more detailed neuropsychological tests to evaluate individual cognitive domains are important to comprehensively differentiate cognitive domain decline. Furthermore, the relatively small number of subjects recruited for the study, and the cross-sectional design of the study, may effectively limit data extrapolation and subsequent application to all PD patients. Thus, future large cohort studies, employing comprehensive cognitive tests, and with long follow-up periods are required to validate our results. In addition, our study demonstrates that plasma Aβ-40 and α-synuclein have sensitivity but lack specificity to discriminate between normal cognitive subjects and PD patients with cognitive impairment. In contrast, T-tau has higher specificity but lower sensitivity for discrimination of normal cognitive subjects and PD patients with cognitive impairment.

## Conclusion

In summary, the results of this study demonstrate that increased plasma α-synuclein, increased plasma T-tau, and decreased plasma Aβ-40 are significantly associated with PD patients with cognitive decline/impairment. Therefore, they could be applied in clinical practice as effective and non-invasive biomarkers for early detection and subsequent monitoring of PD progression.

## Data Availability Statement

The raw data supporting the conclusions of this article will be made available by the authors, without undue reservation, to any qualified researcher.

## Ethics Statement

The studies involving human participants were reviewed and approved by the Institutional review board of Kaohsiung Chang Gung Memorial Hospital (IRB number: 201802352A3B0). The patients/participants provided their written informed consent to participate in this study.

## Author Contributions

All authors listed have made a substantial, direct and intellectual contribution to the work, and approved it for publication. W-CL was project director. C-HL, W-CL, S-YY, and C-CY designed the experiments. C-HL, W-CL, H-LC, S-HL, and Y-HC contributed to the technical expertise contribution. W-CL, N-CC, H-LC, Y-HC, and M-HC drafted the manuscript.

## Conflict of Interest

S-YY was employed by the company MagQu Co., Ltd. The remaining authors declare that the research was conducted in the absence of any commercial or financial relationships that could be construed as a potential conflict of interest.
