# Supplementary material for: Plasma Levels of α-Synuclein, Aβ-40 and T-tau as Biomarkers to Predict Cognitive Impairment in Parkinson’s Disease
Source: Front Aging Neurosci. 2020 Apr 28;12:112. doi: 10.3389/fnagi.2020.00112 (PMC7198695; doi:10.3389/fnagi.2020.00112)
Supplement: Supplementary file 1 [file Presentation_1.pdf]

Supplemental material

Table 1. The correlation between four plasma biomarkers

	α-synuclein	Aβ1-40	Aβ1-42	T-Tau
**α-synuclein**	-	-0.500**	0.406**	0.522**
Aβ1-40	-0.500**	-	-0.678**	-0.841**
Aβ1-42	0.406**	-0.678**	-	0.582**
T-Tau	0.522**	-0.841**	0.582**	-

Spearman rank correlation analysis to determine relationship between plasma biomarkers
** = $p < 0.01$; * =$p < 0.05$

Supplemental material

The variability (intra and inter) of the assay, sensitivity, dilution experiments, parallelism, etc. (all can be found in the Clinical Path Institute guidebook for biomarkers)

The pre-clinical characterizations, such as limit of blank (LoB), limit of detection (LoD), linearity, repeatability, dilution recovery, spiking recovery, and interference test, of assaying these biomarkers were explored according to Clinical and Laboratory Standards Institute (CLSI) guidelines EP5-A3, EP7-A2, EP17-A2, and C28-A2. Results have been published in Refs. A, B and C. The experiments exploring these characterizations are described briefly as follows:

Assay detection limit

The LoB was obtained by determining the appropriate percentile (p) value of the ranked measured concentrations for the blank samples, which was p = 0.95 in this case:
LoB = Results at position [$0.95 \times N_B + 0.5$],

(1) where $N_B = 60$ (N_B is the number of trials) in the current case.
Equation (1) becomes LoB = Results at position 57.5

(2) The LoB was calculated by performing linear interpolation using the 57th- and 58th-ranked measured concentrations.

The limit of detection (LoD) was calculated with the equation
$LoD = LoB + 1.65\sigma_S$

(3) where σ_S denotes the standard deviation of the sixty measured concentrations of a given sample.

Assay linear range

The linear range of the assay is defined according to the biomarker concentrations that reflect the proportionality between the measured concentration and the spiked concentration. The proportional coefficient should be between 0.9 and 1.1, and the coefficient of determination R^2 for the measured-spiked concentration curve should be higher than 0.95. In current cases, more than seven samples with different spiked concentrations were used.

Assay repeatability

The biomarker solutions were measured precisely in one run. Two sequential measurements of two duplicate measurements each were regarded as two runs. Twenty runs were performed in the current case. Two solutions spiked with different biomarker concentrations were used for the tests of reproducibility. By following the statistical method described in the CLSI EP5-A3 guidelines, the within-lab precision and standard deviations of repeatability for biomarker in these two samples were calculated.

Dilution recovery

The dilution recovery is defined as:

$$\text{Dilution recovery} = \frac{\text{measured concentration after dilution}}{\text{expected concentration after dilution}} x100\%$$

(4) The acceptable dilution recovery range is from 90% to 110%. In this work, a biomarker solution with a known concentration was diluted by a factor of between 2 and 100 with PBS solution for exploring the acceptable range of dilution.

Spiking recovery

A sample with a higher biomarker concentration was spiked into another sample with a lower biomarker concentration. The biomarker concentration of the mixture was assayed. The spiked recovery was calculated via

$$\text{Spiked recovery} = \frac{\text{measured concentration after mixing}}{\text{expected concentration after mixing}} x100\%$$

(5) Interference test

A sample of pure PBS solution spiked only with biomarker without any interfering material was assayed. The measured concentration was used as a reference. Other samples with the know biomarker concentration and either of interfering materials, such as 600 μg/ml conjugated bilirubin, 10000 μg/ml hemoglobin, 30000 μg/ml intralipid, 60000 μg/ml albumin, 500 IU/ml rheumatoid factor, 200 μg/ml uric acid, 500 μg/ml acetylsalicylic acid, 300 μg/ml ascorbic acid, 1000 μg/ml ampicillin sodium, 100 μg/ml quetiapine fumarate, 90 ng/ml galantamine hydrobromide, 100 ng/ml rivastigmine hydrogen tartrate, 1000 ng/ml donepezil hydrochloride, and 150 ng/ml memantine hydrochloride, were assayed. The recovery rates in the measured biomarker concentration between the other samples and the reference sample were calculated.
These characterizations of assaying various biomarkers are listed below.

Biomarker	$A\beta_{1-40}$	$A\beta_{1-42}$	T-Tau	α-synuclein
LoB	0.02 pg/ml	0.51 pg/ml	0.01 pg/ml	0.58 fg/ml
LoD	0.170 pg/ml	0.770 pg/ml	0.026 pg/ml	1.396 fg/mL
Linear range	1-1,000 pg/ml	1-30,000 pg/ml	0.1~3,000 pg/ml	0.001396~1,020 pg/ml
Repeatability (CV%)	≤3.7	≤6.4	≤5.6	≤8.6
Within-lab precision (CV%)	≤3.3	≤6.7	≤5.5	≤13.8
Dilution recovery (%)	90.4-104.8 (2-50X)	97.2-108.9 (5-50X)	91.2-108.7 (5-50X)	91.1-97.8 (5-20X)
Spiking recovery (%)	99.2-108.2	94.3-101.9	91.5-101.3	90.0-99.5
Recovery rate of interference test (%)	90.8-109.3	90.1~106.9	91.8~108.9	90.45~109.86

LoB: limit of blank LoD: limit of detection CV: coefficient of variation

[A] Dubois B, Burn D, Goetz C, et al. Yang SY, Chiu MJ, Chen TF, Lin CH, Jeng JS, Tang SC, et al. Analytical performance of reagent for assaying tau protein in human plasma and feasibility study screening neurodegenerative diseases. Sci Reports. 2017;7:9304-1-12.
[B] Teunissen CE, Chiu MJ, Yang CC, Yang SY, Scheltens P , Zetterberg H, Blennow K. Plasma amyloid-β (Aβ42) correlates with cerebrospinal fluid Aβ42 in Alzheimer's disease. J Alz Dise. 2018;62:1857–1863.
[C] Yang SY, Chiu MJ, Lin CH, Horng HE, Yang CC, Chieh JJ, Chen HH, Liu BH. Development of an ultra-high sensitive immunoassay with plasma biomarker for differentiating Parkinson disease dementia from Parkinson disease using antibody functionalized magnetic nanoparticles. J Nanobiotechnol. 2016;14:4-1-8.1
